# Ptomaïnes and Their Effects

**Published:** 1888-12

**Authors:** 


### EDITORIAL.

### PTOMAÏNES AND THEIR EFFECTS.

Ptomaïnes, as is well known, are chemical compounds, the result of putrefaction; but putrefaction comes from the growth of micro-organisms alone. Ptomaïnes are, therefore, formed in no other way than by the growth of such bodies. Their number must be great, greater perhaps than the sorts of germs; for it is not at all unlikely that the same germ in different conditions may produce different ptomaïnes. Already more than forty have been discovered, and their composition determined. Early in their history some of them were found to be highly poisonous, some resembled in their reactions known drugs. Chemists of ability had confounded them with strychnia, morphia, and the like. They were, therefore, from the first, thought to be objects of considerable interest.

The readers of this journal will remember that sometime ago* mention was made of the fact that Brieger had discovered several of these substances in pure cultures of organisms found in tetanus. All of them were highly irritating to the nervous system; with one of them he had produced tetanus.

Tetanus, then, according to him, is caused by bacteria in a secondary way, the bacterium forming the ptomaïne and the ptomaïne producing the symptoms, just as yeast makes alcohol and alcohol causes intoxication.

This discovery added greatly to the interest of these substances. They were now removed from the group of bodies interesting to the chemist and toxicologist alone; they became of importance to the pathologist and practical physician.

Recently, the investigation has been extended. Brieger has found a ptomaïne in cultures of the typhoid bacillus, which causes typhoid symptoms, with ulceration of the agminated glands.

A ptomaïne has been found in cultures of the anthrax bacillus which causes symptoms like those from inoculations of the bacillus itself. There is evidence to show that the cholera bacillus produces a substance highly irritating to the intestinal canal, although the bacilli probably act mechanically also.

An explanation of the way the ptomaïnes act, in some cases, at least, has been given

---

* August, 1887.

by Oertel in an elaborate work on diphtheria just published.

According to him the ptomaïne penetrates the epithelium, is taken up by the wandering white blood corpuscles, and carried to all parts of the body. In their course, these corpuscles are more or less completely destroyed; the blood vessels are injured, and results of the action of the poison are seen, especially, in the kidneys, spleen, and nervous centres. The ptomaïne dissolves first the cell-wall and then the cell itself. By a sort of catalyses, it is reproduced out of the ruins of the destroyed tissues, and the diseased activity continues although the germ from which it originated is destroyed.

It is not at all unlikely that infectious diseases, as a class, have a similar history.

Röhmann, in a recent number of the *Berliner Klinische Wochenschrift*,† makes it, at least, likely that something of this kind occurs in acute atrophy of the liver.

The continuous symptoms in some cases of ptomaïne poisoning, in which articles containing the poison have been cooked and the germ, therefore, destroyed, are probably to be experienced in this way.

The field opened up to us by these studies, as well as that of the analogous leucomaïnes, promises rich results in many ways and is an inviting one for investigators.

† Nos. 43, 44.

